# Supplementary material for: Botanical Collection Patterns and Conservation Categories of the Most Traded Timber Species from the Ecuadorian Amazon: The Role of Protected Areas
Source: Plants (Basel). 2023 Sep 20;12(18):3327. doi: 10.3390/plants12183327 (PMC10536464; doi:10.3390/plants12183327)
Supplement: Supplementary file 1 [file plants-12-03327-s001.zip › plants-2558710-supplementary.pdf]

Table S1. List of the 214 analysed species with number of records and current IUCN category

ID	Specie	Records	IUCN category
1	*Guarea kunthiana*	481	LC
2	*Grias neuberthii*	329	LC
3	*Mayna odorata*	322	LC
4	*Guarea macrophylla*	296	LC
5	*Tapirira guianensis*	287	LC
6	*Dacryodes peruviana*	220	LC
7	*Matisia malacocalyx*	206	LC
8	*Minquartia guianensis*	192	LC
9	*Alchornea glandulosa*	175	LC
10	*Apeiba membranacea*	174	LC
11	*Pseudolmedia laevis*	168	LC
12	*Banara guianensis*	167	LC
13	*Micropholis venulosa*	160	LC
14	*Nectandra membranacea*	154	LC
15	*Guarea guidonia*	152	LC
16	*Symphonia globulifera*	151	LC
17	*Ocotea javitensis*	148	LC
18	*Pseudolmedia laevigata*	147	LC
19	*Piptocoma discolor*	146	LC
20	*Trema micrantha*	146	NE
21	*Terminalia amazonia*	140	LC
22	*Batocarpus orinocensis*	138	LC
23	*Clarisia biflora*	137	LC
24	*Inga edulis*	137	LC
25	*Huertea glandulosa*	134	LC
26	*Caryodendron orinocense*	132	LC
27	*Virola duckei*	132	LC
28	*Ficus maxima*	130	LC
29	*Browneopsis ucayalina*	128	LC
30	*Pourouma minor*	128	LC
31	*Virola pavonis*	127	LC
32	*Pourouma cecropiifolia*	125	LC
33	*Vernonanthura patens*	125	LC
34	*Pouteria caimito*	124	LC
35	*Osteophloeum platyspermum*	122	LC
36	*Cedrelinga cateniformis*	117	LC
37	*Solanum grandiflorum*	117	LC
38	*Virola flexuosa*	117	LC
39	*Celtis schippii*	116	LC
40	*Ladenbergia oblongifolia*	116	LC
41	*Virola sebifera*	110	LC
42	*Cedrela odorata*	108	VU
43	*Turpinia occidentalis*	107	LC

44	*Cordia alliodora*	106	LC
45	*Metteniusa tessmanniana*	106	LC
46	*Brosimum guianense*	103	LC
47	*Cabralea canjerana*	102	LC
48	*Casearia arborea*	102	LC
49	*Vismia baccifera*	102	LC
50	*Clarisia racemosa*	101	LC
51	*Apeiba aspera*	100	NE
52	*Nectandra reticulata*	100	LC
53	*Matisia cordata*	98	LC
54	*Trichilia pleeana*	98	LC
55	*Guarea gomma*	96	LC
56	*Guarea purusana*	91	LC
57	*Pseudolmedia rigida*	89	LC
58	*Heliocarpus americanus*	88	LC
59	*Pouteria multiflora*	88	NE
60	*Abarema jupunba*	87	LC
61	*Terminalia oblonga*	87	LC
62	*Cupania cinerea*	86	LC
63	*Ilex guayusa*	85	LC
64	*Drimys granadensis*	84	NE
65	*Jacaranda copaia*	83	LC
66	*Couroupita guianensis*	82	LC
67	*Myrsine coriacea*	82	NE
68	*Cecropia sciadophylla*	81	LC
69	*Chimarrhis glabriflora*	81	LC
70	*Simarouba amara*	80	LC
71	*Hymenaea oblongifolia*	79	LC
72	*Gustavia macarenensis*	76	LC
73	*Miconia prasina*	76	LC
74	*Brosimum utile*	71	LC
75	*Ochroma pyramidale*	69	LC
76	*Parkia multijuga*	69	LC
77	*Sloanea fragrans*	69	LC
78	*Cestrum racemosum*	68	LC
79	*Calliandra angustifolia*	67	LC
80	*Chrysophyllum argenteum*	66	LC
81	*Vochysia braceliniae*	65	LC
82	*Erythrina poeppigiana*	64	LC
83	*Podocarpus oleifolius*	64	LC
84	*Schefflera morototoni*	63	NE
85	*Triplaris dugandii*	63	LC
86	*Calycophyllum spruceanum*	62	NE
87	*Cespedesia spathulata*	61	LC
88	*Erisma uncinatum*	61	LC
89	*Ceiba samauma*	60	NE

90	*Ceiba pentandra*	59	LC
91	*Poulsenia armata*	58	LC
92	*Boehmeria caudata*	57	LC
93	*Morella pubescens*	57	LC
94	*Calophyllum brasiliense*	54	LC
95	*Genipa americana*	54	LC
96	*Laetia procera*	54	NE
97	*Myroxylon balsamum*	54	LC
98	*Persea americana*	52	LC
99	*Calyptranthes plicata*	50	NE
100	*Trattinnickia glaziovii*	49	LC
101	*Vallea stipularis*	48	LC
102	*Myrcianthes rhopaloides*	47	LC
103	*Capirona decorticans*	46	LC
104	*Protium fimbriatum*	44	NE
105	*Simira cordifolia*	42	LC
106	*Clusia multiflora*	41	LC
107	*Cassia cowanii*	40	LC
108	*Endlicheria gracilis*	39	LC
109	*Aniba riparia*	38	LC
110	*Schizolobium parahyba*	37	LC
111	*Theobroma bicolor*	37	LC
112	*Elaeagia utilis*	35	LC
113	*Rollinia mucosa*	35	NE
114	*Hedyosmum cuatrecazanum*	34	LC
115	*Mauria heterophylla*	34	LC
116	*Sapium stylare*	33	NE
117	*Sloanea grandiflora*	33	LC
118	*Piptadenia pteroclada*	32	LC
119	*Vitex cymosa*	32	LC
120	*Artocarpus altilis*	31	NE
121	*Crescentia cujete*	31	LC
122	*Jacaratia spinosa*	30	LC
123	*Cinchona officinalis*	29	NE
124	*Trema integerrima*	29	NE
125	*Tabebuia chrysantha*	28	NE
126	*Ficus tonduzii standl.*	27	NE
127	*Psychotria brachiata*	27	NE
128	*Croton sampatik*	25	NE
129	*Virola dixonii*	25	NE
130	*Ocotea infrafoveolata*	23	LC
131	*Prunus opaca*	22	LC
132	*Nectandra laurel*	21	LC
133	*Prumnopitys montana*	21	VU
134	*Virola reidii*	21	NE
135	*Pouteria lucuma*	20	LC

136	*Syzygium jambos*	19	LC
137	*Hyeronima alchorneoides*	18	NE
138	*Maclura tinctoria*	18	LC
139	*Persea ferruginea*	18	NE
140	*Triplaris cumingiana*	16	LC
141	*Lafoensia acuminata*	14	LC
142	*Ocotea sericea*	14	LC
143	*Trichanthera gigantea*	14	LC
144	*Persea rigens*	12	LC
145	*Aniba perutilis*	11	VU
146	*Brosimum alicastrum*	11	LC
147	*Physalis peruviana*	11	NE
148	*Acacia glomerosa*	10	NE
149	*Lonchocarpus nicou*	10	NE
150	*Sapindus saponaria*	10	LC
151	*Phytolacca dioica*	9	NE
152	*Platymiscium pinnatum*	9	NE
153	*Aegiphila alba*	8	LC
154	*Euphorbia laurifolia*	8	LC
155	*Rhamnus granulosa*	8	NE
156	*Aegiphila cuatrecasasii*	7	LC
157	*Ficus cuatrecasana*	7	NE
158	*Lonchocarpus araripensis*	7	NE
159	*Mouriri oligantha*	7	LC
160	*Vochysia ferruginea*	7	LC
161	*Carapa guianensis*	6	LC
162	*Styloceras laurifolium*	6	LC
163	*Alseis eggersii*	5	NE
164	*Centrolobium ochroxylum*	5	CR
165	*Otoba gordoniifolia*	5	NE
166	*Pachira rupicola*	5	DD
167	*Vitex gigantea*	5	LC
168	*Ceiba insignis*	4	NE
169	*Dialyanthera parvifolia*	4	NE
170	*Leucaena leucocephala*	4	NE
171	*Prunus serotina*	4	LC
172	*Rheedia edulis*	4	NE
173	*Anacardium excelsum*	3	NE
174	*Dussia lehmannii*	3	LC
175	*Hyeronima oblonga*	3	NE
176	*Matisia coloradorum*	3	EN
177	*Muntingia calabura*	3	NE
178	*Myrcia acuminata*	3	NE
179	*Pleurothyrium tomiwahlii*	3	NE
180	*Bursera graveolens*	2	LC
181	*Caesalpinia spinosa*	2	NE

182	*Caryodendron amazonicum*	2	NE
183	*Castilla tunu*	2	LC
184	*Centrolobium paraense*	2	NE
185	*Cojoba arborea*	2	LC
186	*Geoffroea spinosa*	2	LC
187	*Humiriastrum procerum*	2	VU
188	*Ochroma lagopus*	2	NE
189	*Sapium utile*	2	NE
190	*Tamarindus indica*	2	LC
191	*Weinmannia glabra*	2	NE
192	*Albizia multiflora*	1	NE
193	*Caesalpinia glabrata*	1	NE
194	*Castilla elastica*	1	LC
195	*Cordia lutea*	1	LC
196	*Cordia macrantha*	1	NE
197	*Cynometra bauhiniifolia*	1	LC
198	*Gallesia integrifolia*	1	LC
199	*Guarea cartaguenya*	1	VU
200	*Hyeronima macrocarpa*	1	NE
201	*Jacaranda mimosifolia*	1	VU
202	*Laguncularia racemosa*	1	LC
203	*Myrcianthes hallii*	1	NE
204	*Nectandra guadaripo*	1	VU
205	*Otoba gracilipes*	1	NE
206	*Piscidia carthagenensis*	1	LC
207	*Pseudobombax millei*	1	DD
208	*Swartzia littlei*	1	EN
209	*Tabebuia guayacan*	1	NE
210	*Terminalia valverdeae*	1	NE
211	*Tetragastris varians*	1	NE
212	*Triplaris guayaquilensis*	1	NE
213	*Vochysia macrophylla*	1	LC
214	*Ziziphus thyrsiflora*	1	NE
